# 

**Published:** 1891-03

**Authors:** 


The American Medical Association will hold its forty-second annual meeting in Washington, on Tuesday, Wednesday, Thursday, and Friday, May 5, 6, 7, and 8, 1891.

---

The meeting of National Association of Railway Surgeons will be held in Buffalo, commencing Tuesday, April 30, 1891, and continuing three days.

### BOOKS AND PAMPHLETS RECEIVED.

A Practical Treatise on Fractures and Dislocations. By Frank Hastings Hamilton, A. B., A. M., M. D., L. L. D., Late Professor of Surgery in Bellevue Hospital Medical College, and Surgeon to Bellevue Hospital, New York; Consulting Surgeon to Hospital for Ruptured and Crippled, to St. Elizabeth's Hospital, etc.; author of a Treatise on Military Surgery and Hygiene; a Treatise on the Principles and Practice of Surgery, etc. Eighth edition, revised and edited by Stephen Smith, A. M., M. D., Professor of Clinical Surgery in the University of the City of New York and Surgeon to Bellevue and St Vincent's Hospitals, New York. Illustrated with 507 wood-cuts. Pp. xvi.—849. Philadelphia: Lea Brothers & Co. 1891. Price, cloth, $5.50; sheep, $6.50.

A Compend of Diseases of Children, especially adapted for the use of medical students. By Marcus P. Hatfield, A. M., M. D., Professor of Diseases of Children, Chicago Medical College; Physician to Wesley Hospital, etc. With a colored plate. Quiz Compends No. 14. Philadelphia: P. Blakiston, Son & Company. 1890.

A Guide to the Practical Examination of Urine. For the use of physicians and students. By James Tyson, M. D., Professor of Clinical Medicine in the University of Pennsylvania, and Physician to the Hospital of the University; Fellow of the College of Physicians of Philadelphia, etc., etc. Seventh edition, revised and corrected. With a colored plate and wood engravings. Philadelphia: P. Blakiston, Son & Company, 1891.

Manual of Clinical Diagnosis. By Dr. Otto Seifert, Privatdocent in Würzburg, and Dr. Friedrich Müller, Assistent der II. Med. Klinik in Berlin. Translated from the fifth German edition, enlarged and revised with the permission of the authors. By William Buckingham Canfield, A. M., M. D., (Berlin) Fellow of the American Academy of Medicine, Member of the Medical and Chirurgical Faculty of Maryland; Visiting Physician to the Union Protestant Infirmary of Baltimore; Lecturer on Clinical Medicine, and Chief of Chest Clinic University of

Maryland. Second English edition, revised and enlarged, with fifty illustrations and one colored plate. New York and London: G. P. Putnam's Sons. The Knickerbocker Press. 1890.

Études Sur La Rage et la méthode Pasteur. Par le Dr. Lutaud, Rédacteur en chef du *Journal de Médecine* de Paris. Second edition, pp. 440, 12mo. Paris: *Journal de Médicine*, 35 Boulevard Haussman. 1891.

Principles of Surgery. By N. Senn, M. D., Ph. D., Milwaukee, Wis. Professor Principles of Surgery and Surgical Pathology in the Rush Medical College, Chicago, Ill.; Professor of Surgery in the Chicago Polyclinic; Attending Surgeon to the Milwaukee Hospital, etc. Illustrated with 109 wood engravings. Octavo, pp. xiii. – 611. Philadelphia and London: F. A. Davis. 1890. Price, cloth, $4.50; sheep, $5.50 net.

Heredity, Health and Personal Beauty. By John V. Shoemaker, A. M., M. D.; Professor of Materia Medica and Pharmacology, Therapeutics and Clinical Medicine, and Clinical Professor of Diseases of the Skin in the Medico-Chirurgical College of Philadelphia; Physician to the Medico-Chirurgical Hospital, etc. Octavo, pp. xv.—422. Philadelphia and London: F. A. Davis. 1890. Price, cloth, $2.50; ½ Mor., $3.50 net.

Transactions of the American Gynecological Society. Vol. XV. For the year 1890. Small octavo, pp. xxxix.—411. Philadelphia: William J. Dornan. 1890.

Transactions of the American Association of Obstetricians and Gynecologists. Vol. III., for the year 1890. Standard 8vo, pp., xliii.—385. Illustrated with nineteen plates, two charts, and forty-six engravings. Philadelphia: William J. Dornan. 1891.

Wood's Medical and Surgical Monographs. Vol. IX., No. 1. January, 1891. Published monthly. New York: William Wood & Company. Price, $10.00 a year; single copies, $1.00.

I. Retroversion and Retroflexion of the Gravid Uterus. II. Some Points in the Technique of Laparatomy. III. A Series of Cases of Abdominal Section. (Reprinted from the *Edinburgh Medical Journal.*) By J. Halliday Croom, M. D., F. R. C. P. E., F. R. S. E., Physician and Clinical Lecturer on Diseases of Women, Royal Infirmary, Physician Royal Maternity Hospital, etc.

Laws of the State of Michigan Relating to the Public Health in Force in the Year 1890. Compiled under the direction of the Secretary of the State Board of Health. Lansing: Darius D. Thorp, State Printer and Binder. 1889.

Cornell University College of Agriculture. Bulletin of the Agricultural Experiment Stations, All Divisions, XXV.; December, 1890. Sundry Investigations made during the year. Ithaca: Published by the University. 1890.

Proceedings and Addresses at a Sanitary Convention held at Lapeer, Mich., March 27 and 28, 1890. Supplement to the Report of the Michigan State Board of Health for the year 1890.

Phenacatine-Bayer: Its Action; Recent Results. Wm. H. Schieffelin & Co. New York: 1890.

Annual Report of the Postmaster-General of the United States for the fiscal year ending June 30, 1890. Washington: Government Printing Office. 1890.

Mechanical Obstruction in Diseases of the Uterus. By George F. Hulbert, M. D. St. Louis: Reprint, *The Medical News*, December 20, 1890.

Use and Abuse of the Obstetrical Forceps. By Eugene Prosper Bernardy, M. D., Philadelphia. Reprint, Transactions American Association of Obstetricians and Gynecologists. Vol. III. 1890.

Twenty-Second Annual Report of the New York Physicians' Mutual Aid Association, By-Laws and List of Members. New York: William Wieser, 1891.

Nasal Intubation. By D. H. Goodwillie, M. D. Reprint, *The New York Medical Journal*, May 17, 1890.

### HEALTH BULLETINS.

New York State Board of Health for December, 1890.

Tennessee State Board of Health, January 20, 1891.

Abstract of Sanitary Reports U. S. Marine Hospital Service. Vol. VI., Nos. 5, 6, 7, and 8.

284 Franklin Street, Buffalo, N. Y.

Notice to Contributors. — We are glad to receive contributions from every one who knows anything of interest to the profession. Articles designed for publication in the Journal should be handed in before the first day of the month. The Editors are not responsible for the views or opinions of contributors. All communications should be addressed to

